# Red Flags for Chronic Inflammatory Demyelinating Polyradiculoneuropathy Associated with Sarcoidosis or Connective Tissue Diseases

**DOI:** 10.3390/jcm12093281

**Published:** 2023-05-04

**Authors:** Clément Vialatte de Pémille, Nicolas Noël, Clovis Adam, Céline Labeyrie, Adeline Not, Guillemette Beaudonnet, Andoni Echaniz-Laguna, David Adams, Cécile Cauquil

**Affiliations:** 1 Neurology Department, AP-HP, CHU de Bicêtre, 78 Rue du General Leclerc, 94270 Le Kremlin Bicetre, France; 2 Internal Medicine Departement, CHU de Bicêtre, 78 Rue du General Leclerc, 94270 Le Kremlin Bicetre, France; 3 Faculty of Medicine, Paris Saclay University, 63 Rue Gabriel Peri, 94270 Le Kremlin Bicetre, France; 4 Pathology Laboratory, CHU de Bicêtre, 78 Rue du General Leclerc, 94270 Le Kremlin Bicetre, France; 5 French National Reference Center for Rare Neuropathies (NNERF), CHU de Bicêtre, 78 Rue du General Leclerc, 94275 Le Kremlin Bicetre, France; 6 Neurophysiology Department, AP-HP, CHU de Bicêtre, 78 Rue du General Leclerc, 94270 Le Kremlin Bicetre, France; 7 INSERM U1195, Paris Saclay University, 94276 Le Kremlin Bicetre, France

**Keywords:** chronic inflammatory demyelinating polyradiculoneuropathy, neurosarcoidosis, connective tissue disease, Sjögren’s syndrome, therapy

## Abstract

Chronic inflammatory demyelinating polyradiculoneuropathy (CIDP) is a rare autoimmune disorder of the peripheral nervous system. Diagnosis relies on clinical and electrophysiological criteria. Various disorders requiring specific treatment regimens may be associated with CIDP, including sarcoidosis (SAR-CIDP) and connective tissue disease (CTD-CIDP). Therefore, it is important to distinguish between CIDP, SAR-CIDP and CTD-CIDP. In this retrospective monocentric study, we analyzed 16 patients with SAR-CIDP and 11 with CTD-CIDP and compared them with a group of 17 patients with idiopathic CIDP. SAR-CIDP patients had a frequently acute or subacute CIDP onset. CTD-CIDPs were mostly Sjögren’s syndrome and lupus, and patients had a chronic onset. An older age at onset (64.5 vs. 54 years, *p* = 0.04), more atypical presentation (19/25 (76%) vs. 6/17 (35%), *p* = 0.008), acute/subacute onset of symptoms (15/25 (60%) vs. 1/17 (6%), *p* = 0.0004) and more frequent weight loss (7/16 (44%) vs. 0/17 (0%), *p* = 0.017) were identified SAR-CIDP and CTD-CIDP groups. Response to intravenous immunoglobulin therapy was lower in the combined SAR-CIDP and CTD-CIDP group (44% versus 82%, *p* = 0.005). As sarcoidosis and CTDs might be associated with CIDP and require specific management, the “red flags” mentioned above should be kept in mind by clinicians managing patients with CIDP.

## 1. Introduction

Chronic inflammatory demyelinating polyradiculoneuropathy (CIDP) is an autoimmune disorder affecting the peripheral nerves and is typically characterized by symmetrical proximal motor weakness and sensory dysfunction affecting proprioception, associated with areflexia. The diagnosis is based on clinical presentation and electrophysiological data. Nerve conduction studies (NCSs) are crucial for the diagnosis, according to the European Federation of Neurological Societies-Peripheral Nerve Society (EFNS-PNS) criteria [1]. Based on these criteria, so-called supportive diagnostic tools may be necessary for the diagnosis of CIDP: raised cerebrospinal fluid (CSF) protein level without pleiocytosis, inflammatory pattern of nerve roots and/or plexus on magnetic resonance imaging (MRI) and response to immunomodulatory treatment. Despite these supportive diagnostic tools, the diagnosis of CIDP might remain challenging, and other diagnoses must be ruled out [1].

Sarcoidosis is a systemic inflammatory disorder in which peripheral nervous system (PNS) involvement is rare, representing approximately 14% of all neurological involvements [2]. Within the entities of PNS involvement in sarcoidosis, CIDP is a rare feature that may mimic idiopathic CIDP in the absence of previously known sarcoidosis [2].

Connective tissue diseases (CTDs) are autoimmune disorders that affect various organs. The most frequent CTDs consist of rheumatoid arthritis (RA), Sjögren's syndrome (SS), systemic lupus erythematous (SLE) and systemic sclerosis. PNS involvement in SS and SLE has been reported in 7% and 10% of cases, respectively [3,4], and the most common clinical patterns are sensory neuronopathy and mononeuritis multiplex. However, CIDP has rarely been described.

In patients presenting CIDP, it might be thus challenging to distinguish between idiopathic CIDP and that associated with systemic disorders such as sarcoidosis or CTDs. This is of relevance due to the impact on therapeutic decisions for the patients.

In our study, we analyzed a series of patients with CIDP associated with sarcoidosis (SAR-CIDP) and CIDP associated with CTD (CTD-CIDP) and compared them to a group of patients with idiopathic CIDP in order to identify the features pointing to diagnose an associated sarcoidosis or a CTD in the setting of CIDP.

## 2. Materials and Methods

### 2.1. Protocol Approval and Patient Consent

This study was accepted and achieved under the ethical guidelines issued by our institutions for clinical studies. This retrospective study was in compliance with the tenets of the Helsinki Declaration. All patients gave verbal informed consent. In our study, only retrospective medical data obtained during the care were analyzed in conformity with the French Jardé law and the Greater Paris University Hospitals’ (AP-HP) requirements.

### 2.2. Patients

We performed a retrospective monocentric study in a French national reference center for rare polyneuropathies. Data collection ranged from 1989 to 2021. Patients were identified using a local database for inflammatory neuropathy and sarcoidosis or CTD. We included patients with definite, probable, possible or undefined CIDP depending on NCS studies, and the aforementioned support criteria according to EFNS-PNS criteria [1].

Inclusion criteria: all consecutive patients followed in our reference center for both CIDP+SARCOIDOSIS (SAR-CIDP group, n = 17) and CIDP+CONNECTIVE TISSUE DISEASE (CTD-CIDP, n = 11) were included via Assistance Publique-Hôpitaux de Paris APHP (APHP) open source software i2B2 using MeSH terms. There were no exclusion criteria.

Neurosarcoidosis (NS) was classified as definite (granuloma found within the nervous tissue), probable (granuloma found elsewhere and neurological disorders) or possible (no granuloma found but compatible neurological disorders) according to Zajicek criteria [2].

The connective tissue diseases were defined according to the American College of Rheumatology (ACR) criteria for systemic lupus erythematosus (SLE), Sjögren’s syndrome (SS) or mixed connective tissue disease [5,6].

The patients were divided into two groups according to the final diagnosis: CIDP-associated with sarcoidosis (SAR-CIDP) and CIDP-associated with CTD (CTD-CIDP). The control group (idiopathic CIDP) included consecutive patients recruited from our outpatient clinic, all fulfilling definite EFNS-PNS CIDP diagnosis [1].

Typical CIDP was defined as progressive (>8 weeks) distal sensory and proximal motor impairment with areflexia [1]. Atypical CIDP was defined as either asymmetrical CIDP, pure motor neuropathy, distal acquired demyelinating symmetric (DADS) neuropathy or Lewis–Sumner syndrome (upper limb involvement) [1].

### 2.3. Electrophysiological Studies

Electrodiagnostic studies were performed including upper- and lower-limb nerve conduction studies (NCSs) and electromyography (EMG) using a concentric needle electrode in at least 2 muscles. These analyses were performed by a unique operator (GB). Studies were achieved using regular equipment and accepted methods, and skin temperature was kept in the 32–34 °C range.

### 2.4. Data Collection

The following data were collected retrospectively according to the medical file of the patients: demographics (age at onset, sex), clinical presentation and neurological/systemic examination at baseline and outcome (topography, type of onset: acute/subacute or chronic as defined by [1]), cranial nerve involvement and biological data (including autoimmunity, C3/C4 levels, renal or hepatic involvement for CTD or sarcoidosis, Angiotensin I converting enzyme (ACE) serum levels and the existence of a monoclonal gammopathy). Supportive diagnostic tools included CSF findings, nerve root MRI findings and nerve and muscle biopsy findings and were analyzed. Nerve and muscle biopsies were performed according to recommendations at the time of the examination, mostly due to poor response to a first line regiment of intravenous immunoglobulin (IVIG) or suspicion of nerve vasculitis.

### 2.5. Statistics

Statistical analysis was performed using the R language (version 3.2.3, 2015-12-10). According to the type and distribution of the variables we used the non-parametric Wilcoxon test, Student T test, Chi2 test or Fisher exact test. When necessary, multiple testing correction was performed using the Benjamini–Hochberg method. Graphics were produced using the ggplot2 R package. A *p* value under 0.05 was considered significant.

## 3. Results

### 3.1. Cohort Description and Control Group (Figure 1 and Table 1)

**Figure 1:**
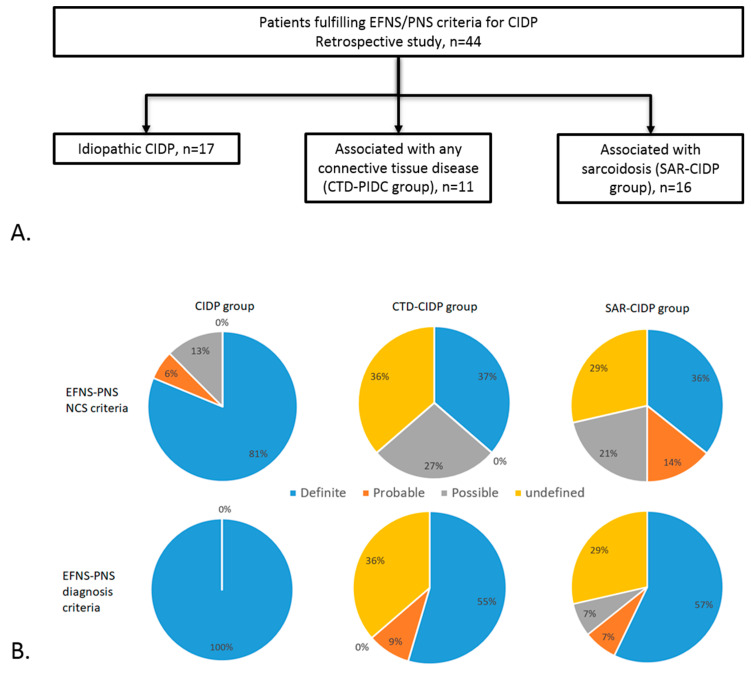
(**A**) Flowchart of the study. (**B**) Proportion of patients fulfilling the EFNS/PNS NCS criteria (**top**) and the EFNS/PNS diagnosis criteria among the three groups of patients.

A total of 44 patients (23 women, median age at symptom onset 60 yo, interquartile range (IQR) of 50–68 yo), were included in the study: 17 with CIDP, 16 with SAR-CIDP and 11 with CTD-CIDP (Figure 1).

#### 3.1.1. SAR-CIDP Group

The median age at neurological onset was 66 yo [IQR 56–73], with 44% of patients being women. All SAR-CIDP patients but one had histologically proven granuloma. There were nine definite peripheral neurosarcoidosis (NS) (9/13 positive nerve biopsies), six probable NS and one possible NS according to Zajicek criteria. The localizations of the granuloma defining the sarcoidosis are summarized in Table 2. Ten had NS diagnosis within one year and three had one between one and three years after neurological onset. In two patients (12.5%), sarcoidosis was already diagnosed at its neurological onset.

Among SAR-CIDP patients, 14 NCSs were available. The patients were classified as having definite (n = 5), probable (n = 2), possible (n = 3) and undefined (n = 4) CIDP according to EFNS-PNS criteria. After correction for supportive criteria, they were classified as definite PIDC in eight cases, possible in one, probable in one and undefined in four (Figure 1).

When available (n = 14), symptoms at onset were: acute with relapses in 5/14 (36%) cases, subacute in 6/14 (43%) cases and chronic in 3/14 (21%) cases with an atypical presentation in 14/14 patients. Facial palsy occurred in 4/11 cases and accounted for 4/5 cranial nerve involvements.

CSF pleiocytosis was observed in 3/10 (30%) cases and elevated CSF protein levels in 8/11 (73%) cases. Angiotensin-I-converting enzyme (ACE) serum levels were elevated in 2/10 (20%) cases and hypercalcemia was found in 1/8 (12%) cases. C-reactive protein was elevated at diagnosis in 3/8 (37%) cases. No patients had monoclonal gammopathy (0/10) and 3/10 (30%) had polyclonal hypergammaglobulinemia. Extra-neurological involvement was present in 8/16 patients (50%) at diagnosis (Table 3).

Nerve biopsy results (n = 13) showed a demyelinating pattern in 9/13 (69%) patients, among which four showed teasing fiber processes. In nine cases, the granuloma was identified within the nerve. Specific localizations were the epineurium (6/8, 75%) and endoneurium (5/8, 62%). Among the twelve muscle biopsies performed, granulomas were found in nine (75%), with two cases of concomitant negative nerve biopsy (Table 2). The minor salivary gland biopsy (MSGB) disclosed granuloma in 2/7 patients, including one in whom nerve biopsy was not performed (Table 2). Concerning therapeutic responses, intravenous immunoglobulin (IVIG) was effective in 1/7 (14%) patients while steroids were effective in 13/13 patients. Plasmapheresis was inefficient when tested (0/1). Four patients had received another immunomodulatory drug during their medical history of sarcoidosis.

#### 3.1.2. CTD-CIDP Group

The median age at neurological onset was 62 yo [IQR 53–67], with 82% being women. Diagnoses were SS (n = 7), SLE (n = 1), SLE associated with SS (n = 2) and mixed connective tissue diseases (n = 1). Three patients had CTD diagnosed within one year and four patients between 1 and 15 years after neurological onset. In four patients (36%), the CTD was already diagnosed at neurological onset.

NCSs classified patients as having definite (n = 4), possible (n = 3) and undefined (n = 4) CIDP according to EFNS-PNS criteria. After correction for supportive criteria, they were classified as having definite CIDP in six cases, probable in one and undefined in four (Figure 1).

Symptom onset was acute with relapses in 2/11 patients (18%), subacute in 2/11 (18%) and chronic in 7/11 (64%). When the data were reported, cranial nerve involvement occurred in 2/6 patients (33%, facial and trigeminal nerve).

Extra-neurological involvement was present in 9/11 patients (82%) at diagnosis (Table 3). C3 and C4 complement fractions were low in 1/9 (11%) and 5/9 (55%) of patients, respectively. C-reactive protein level was elevated at diagnosis in 1/10 patients. MSGB was performed in ten patients and revealed a Chisholm score above three in seven patients (70%). CSF disclosed lymphocytic pleiocytosis in 1/8 (12%) patients and elevated protein level in 5/10 (50%) patients. Nerve biopsy results (n = 4) showed a demyelinating pattern in 2/4 cases (50%).

Concerning the therapeutic response, IVIGs were effective in 7/11 (64%) patients, while steroids were effective in 5/5 patients when used. Five patients had received an immunomodulatory drug during their medical history, but this effect on the CIDP could not be evaluated.

#### 3.1.3. Control Group

The median age was 54 yo [IQR 48–60] at onset with 41% being women. Clinical presentations were typical in 11/17 (65%) and atypical in 6/17 (35%) patients, consisting of purely sensory CIDP (6/6). Responses to therapeutics represented 14/17 patients (82%) for IVIG regiment, 2/2 (100%) for plasmapheresis and 4/4 (100%) for corticosteroid use.

### 3.2. Comparison between SAR-CIDP and CIDP (Table 1)

**Figure 2:**
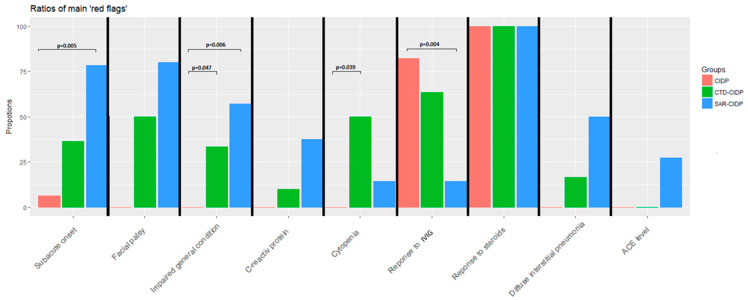
Proportion of red flags among the three groups of patients.

In the SAR-CIDP group, we found statistically more atypical presentation of the CIDP (see definitions in the Materials and Methods section and [1]) (14/14 (100%) vs. 6/17 (35%), *p* = 0.0001), weight loss (4/7 (57%) vs. 0/14 (0%), *p* = 0.006) and acute/subacute onset (11/14 (79%) vs. 1/17 (6%), *p* = 0.0002) (Figure 2). The frequency of facial palsy was similar. NCS patterns did not differ between SAR-CIDP and CIDP groups, with motor conduction blocks found in 47% vs. 83% (*p* = 0.12) of patients, respectively, and only 20% (*p* = 0.01) in the CTD-CIDP group. IVIG response was statistically lower in the SAR-CIDP group (1/7 (14%) vs. 14/17 (82%), *p* = 0.007). No other differences were found, especially concerning histological data.

### 3.3. Comparison between CTD-CIDP and CIDP

Weight loss (3/9 (33%) vs. 0/17 (0%), *p* = 0.047) and cytopenia (5/10 (50%) vs. 0/17 (0%), *p* = 0.032) were more frequent in CTD-CIDP (Figure 2). The rates of responses to IVIG and steroids were similar in the two groups.

### 3.4. Other Comparisons

Atypical neurological presentations were more frequent in the SAR-CIDP group than in the CTD-CIDP group (respectively, 14/14 (100%) and 5/11 (45%), *p* = 0.007).

We analyzed non-idiopathic versus idiopathic CIDP through pooling SAR-CIDP and CTD-CIDP (SAR-CTD-CIDP). We found that an older age at onset (64.5 vs. 54 years, *p* = 0.04), more atypical presentation (19/25 (76%) vs. 6/17 (35%), *p* = 0.008), acute/subacute onset of symptoms (15/25 (60%) vs. 1/17 (6%), *p* = 0.0004) and more frequent weight loss (7/16 (44%) vs. 0/17 (0%), *p* = 0.017) identified the SAR-CTD-CIDP group as compared with idiopathic CIDP. Response to IVIG treatment was lower in the SAR-CTD-CIDP group (44% versus 82%, *p* = 0.005), but steroids were effective in all cases.

## 4. Discussion

The diagnosis of CIDP can be challenging because of atypical neurological presentation [1] and the need to exclude differential or associate diagnoses. The treatment of idiopathic CIDP relies on immunomodulation with IVIG, steroids or plasmapheresis. Atypical features (“red flags”) such as unresponsiveness to treatment, general symptoms, cytopenia and rapid evolution should prompt clinicians to consider an underlying diagnosis of CIDP [1,7]. Indeed, CIDP may be associated with other diseases, such as sarcoidosis or CTD, that modify disease management.

Sarcoidosis of the PNS is very rare, since our experience found sarcoid granulomas in 11 cases out of 3475 nerve biopsies throughout 11 years in our tertiary center [8]. Reviewing the literature, SAR-CIDP has been reported in 12 cases of chronic CIDP, and 15 cases of acute inflammatory demyelinating polyradiculoneuropathy (AIDP) [8,9,10,11,12,13,14,15,16,17,18,19,20,21,22,23,24] with only 4 definite NS cases [8,11]. CTD-CIDP has been reported in only 25 cases in the literature [25,26,27,28,29,30,31,32,33,34,35,36,37,38,39,40,41], with the largest description being about 6CIDP associated with SLE [39]. Thus, strictly defined CIDP associated with sarcoidosis or CTD are rare and form only a small range of peripheral nerve involvement in these diseases.

The aim of our study was to describe distinctive features that may help identify associated sarcoidosis or CTD in CIDP patients, which has never been done. We identified several distinctive features (red flags): more frequent weight loss, unresponsiveness to IVIG therapy and acute onset of symptoms in SAR-CIDP. CTD-CIDP patients had more frequent weight loss and cytopenia. Interestingly, CSF protein levels and other biological findings, including ACE levels and calcemia, did not differ between groups which is consistent with previous studies. Extra-neurological involvements were more frequent, as expected. Thus, we recommend a comprehensive clinical examination and a larger work-up including thoracic X-ray, serum protein electrophoresis, auto-antibody testing and accessory salivary gland biopsy in case of the aforementioned red flags. In our study, MSGB, considered as minimally invasive, revealed granuloma in 29% (2/7) of SAR-CIDP patients, which is close to findings regarding the diagnostic accuracy of MSGB in sarcoidosis [42]. In the CTD group, the Chisolm score was consistent with Sjögren’s syndrome in 7/10 patients. Globally, MSGB contributed to diagnosis in more than 50% of cases.

Interestingly, SAR-CIDP and CTD-CIDP patients fulfilled definite EFNS/PNS CIDP diagnosis criteria in more than 50% of cases (respectively, 54% and 57%) and motor conduction blocks were equally found in both the SAR-CIDP and CIDP groups (47% vs. 83%, respectively, (*p* = 0.12). These results are consistent with the literature [8,11] with the hypothesis of a mechanical action of the granuloma in a nerve conduction bloc [11]. However, we found specific demyelinating lesions within 9/12 (69%) of the nerve biopsies of the SAR-CIDP group, and one patient had a characteristic “onion bulb”. These features were distinct from the granulomas’ locations, which suggests a primary demyelinating process in SAR-CIDP. Surprisingly, there were no differences in nerve biopsies between SAR-CIDP and CIDP concerning the inflammatory component either in its quantitative or qualitative features. Finally, associated muscle biopsy increased the sensitivity of nerve biopsy since granuloma findings increased from 69% to 87% of cases (Table 2).

Concerning the response to treatment, our results showed a good response to classic treatment regimen with IVIG use in CTD-CIDP patients on the one hand, whereas on the other hand, use of corticosteroids was needed in SAR-CIDP patients due to resistance to IVIG. One explanation could be the sarcoid granuloma’s involvement in nerve demyelination, either via mechanical component or via a paracrine effect.

Our study has several limitations. Due to the retrospective nature of the data collection, we cannot rule out bias in the selection or the interpretation of some clinical parameters. Additionally, we were unable to collect the exact cumulative dose of corticosteroids in the SAR and CTD groups to evaluate their precise impact. Further, we could not analyze the precise effect of other immunomodulatory drugs in these patients.

## 5. Conclusions

In conclusion, although SAR-CIDP and CTD-CIDP share some common features with CIDP, our study suggests that acute onset, cytopenia, weight loss and non-response to IVIG treatment are good criteria that may indicate the existence of an underlying inflammatory disease in a patient with CIDP. As sarcoidosis and CTDs require specific management, these red flags should be kept in mind by clinicians managing patients with CIDP.

## Figures and Tables

**Table 1:** Statistical comparison between SAR-CIDP, CTD-CIDP and CIDP groups.

		Total (n = 44)	SAR-CIDP (n = 16)	CTD-CIDP (n = 11)	CIDP (n = 17)
**Age of onset, median [Q1–Q3]**		60 * [50–68]	66 [56–73]	62 [53–67]	54 [48–60]
**Sex (%F)**		52	44	82	41
**Atypical presentation, n (%)**		25 * (60%)	14 (100%)	5 (45%)	6 (35%)
CIDP diagnosis according to NCS (definite, probable, possible, undefined, n (%)) *^,¤,†^					
	**Definite**	22 (54%)	5 (36%)	4 (36%)	13 (81%)
	**Probable**	3 (7%)	2 (14%)	0	1 (6%)
	**Possible**	8 (19%)	3 (21%)	3 (27%)	2 (13%)
	**Undefined**	8 (19%)	4 (29%)	4 (36%)	0
CIDP diagnosis after supportive criteria (definite, probable, possible, undefined, n (%)) *^,¤,†^					
	**Definite**	30 (73%)	8 (57%)	6 (55%)	16 (100%)
	**Probable**	2 (5%)	1 (7%)	1 (9%)	0
	**Possible**	1 (2%)	1 (7%)	0	0
	**Undefined**	8 (20%)	4 (29%)	4 (36%)	0
**Weight loss, n (%)**		7 *^,¤,†^ (24%)	4 (57%)	3 (33%)	0
**Extra neurological involvement (n,%)**			8 (50%)	9 (82%)	
**Acute or subacute onset, n (%)**		16 *^,†^ (38%)	11 (79%)	4 (36%)	1 (6%)
**CSF pleiocytosis, n (%)**		5 (19%)	3 (30%)	1 (13%)	1 (12%)
**CSF protein level g/L, median [Q1–Q3]**		0.62 [0.5–1.1]	0.64 [0.6–0.9]	0.41 [0.4–0.7]	0.76 [0.5–1.1]
**Presence of motor conduction blocs on NCS**		19 (51%)	7 (47%)	2 (20%)	10 (83%)
**Response to treatment**					
	**Steroids (n,%)**	22 (100%)	13 (100%)	5 (100%)	4 (100%)
	**IVIG (n,%)**	22 *^,†^ (63%)	1 (14%)	7 (64%)	14 (82%)
	**Plasmapheresis (n,%)**	2 (67%)	0/1	Not prescribed	2 (100%)

Significant comparison (*p* < 0.05): * for SAR-CIDP vs. CIDP, ¤ for CTD-CIDP vs. CIDP and ^†^ SAR-CTD-CIDP vs. CIDP.

**Table 2:** Granuloma locations within tissues in the SAR-CIDP group.

Patient	Tissue with Granuloma	Other Tissue with Granuloma	Zajicek Criteria	Nerve Biopsy	Granuloma in Nerve Biopsy	Muscle Biopsy	Granuloma in Muscle Biopsy	Granuloma in Accesory Salivatory Glands
1			Possible	X	No	X	No	No
2	lung		Probable	X	No	X	No	--
3	nerve	muscle	Definite	X	Yes	X	Yes	Yes
4	muscle		Probable	--		X	Yes	No
5	skin		Probable	--		--		Yes
6	nerve		Definite	X	Yes	X	No	No
7	muscle		Probable	--		X	Yes	No
8	muscle		Probable	X	No	X	Yes	--
9	nerve	muscle	Definite	X	Yes	X	Yes	--
10	nerve		Definite	X	Yes	--		--
11	nerve	muscle, stomac	Definite	X	Yes	X	Yes	--
12	muscle		Probable	X	No	X	Yes	No
13	nerve	muscle	Definite	X	Yes	--		--
14	nerve	muscle	Definite	X	Yes	X	Yes	--
15	nerve		Definite	X	Yes	X	Yes	NP
16	nerve		Definite	X	Yes	--		--

X: performed. --: not performed. Yes: present. No: not present.

**Table 3:** Extra-neurologic involvement in SAR-CIDP and CTD-CIDP groups.

Extra-Neurological Involvement	SAR-CIDP		CTD-CIDP	
	Type	Nb	Type	Nb
Total	50%		82%	
**Lung**	Intersitial pneumonitis	3	DIP	3
**Cardiac**	Pericarditis	1	Pericarditis	2
**Adenopathy**	Thoracic	3		
**Skin**	*Erythema nodosum*	1	*Livedo*	2
	Scar modification	1		
	Nail involvement	1		
**Ocular**	Uveitis	1		
	Episcleritis	1		
**Digestive tract**	Gastritis	1	Hepatitis	1
			Oesophagus hypomotility	1
**Sicca**			7	
**Rheumatologic**			Arthralgia	3
**Raynaud syndrome**			2	
**Thyroiditis**			2	
**Hematologic**			Cytopenia	1
			Myelofibrosis	1
**Muscle**			Myositis	1

## Data Availability

Data are available upon request by the corresponding author.
